# “Listen to Your Immune System When It’s Calling for You”: Monitoring Autoimmune Diseases Using the iShU App

**DOI:** 10.3390/s22103834

**Published:** 2022-05-18

**Authors:** Cláudia Ortet, Liliana Vale Costa

**Affiliations:** Department of Communication and Art—DigiMedia, University of Aveiro, 3810-193 Aveiro, Portugal

**Keywords:** wearable sensors, gamification, self-care, autoimmune disease management, inflammation surveillance, mHealth

## Abstract

The immune system plays a key role in protecting living beings against bacteria, viruses, and fungi, among other pathogens, which may be harmful and represent a threat to our own health. However, for reasons that are not fully understood, in some people this protective mechanism accidentally attacks the organs and tissues, thus causing inflammation and leads to the development of autoimmune diseases. Remote monitoring of human health involves the use of sensor network technology as a means of capturing patient data, and wearable devices, such as smartwatches, have lately been considered good collectors of biofeedback data, owing to their easy connectivity with a mHealth system. Moreover, the use of gamification may encourage the frequent usage of such devices and behavior changes to improve self-care for autoimmune diseases. This study reports on the use of wearable sensors for inflammation surveillance and autoimmune disease management based on a literature search and evaluation of an app prototype with fifteen stakeholders, in which eight participants were diagnosed with autoimmune or inflammatory diseases and four were healthcare professionals. Of these, six were experts in human–computer interaction to assess critical aspects of user experience. The developed prototype allows the monitoring of autoimmune diseases in pre-, during-, and post-inflammatory crises, meeting the personal needs of people with this health condition. The findings suggest that the proposed prototype—iShU—achieves its purpose and the overall experience may serve as a foundation for designing inflammation surveillance and autoimmune disease management monitoring solutions.

## 1. Introduction

One of the primary characteristics of the immune system is its ability to discriminate between self and non-self-antigens. This unique feature is “understood” by previously educated lymphocytes capable of recognizing and responding to foreign antigens and not responding to self-antigens. Indeed, the nonresponsiveness of the cells of the immune system against self-antigens refers to immunological tolerance, whereas autoimmunity is related to the loss of control of the mechanisms that maintain tolerance.

In a broader sense, autoimmunity consists of the immune responses system of an organism which acts against its own (healthy) cells, tissues, organs, and other body normal constituents. Any disease caused by this type of immune response is an autoimmune disease, which can result from the persistent loss of control mechanisms responsible for maintaining tolerance to self-antigens, and the symptoms depend on the part of the body that is affected, even if the common signs are the typical symptoms of inflammation [1]. In autoimmune diseases, different treatment approaches can be used to achieve remission or at least alleviate symptoms, with wearable sensors being a personalized aid technology that can contribute to disease management.

Currently, sensors for personal use (e.g., wearables) have been widely recognized to facilitate lifestyle monitoring, as these often provide diverse information about the user, giving a basic notion of the common physiological signs in certain activities [2]. In addition, this type of technology is becoming omnipresent in citizens’ lives, being also applied to health monitoring, involving medical supervision and patients’ data [3].

In fact, wearable technologies are almost exclusively applied in the health sector and can be an important tool in reducing the time of medical diagnoses, helping to combat the waste of resources involved, as well as promoting human health and wellbeing. On the one hand, from the perspective of customary health care intervention, there are five main qualities of wearable devices [4]: (i) wireless movability; (ii) interactivity and intelligence; (iii) sustainability and durability; (iv) easy operation and miniaturization; and (v) wearability and transportability. On the other hand, from the perspective of modern medicine, the application of wearable devices in the field of health follows the 4P medical model (i.e., preventive, predictive, personalized, and participatory medicine) [5].

The forms of treatment and diagnoses through devices sensitive to the parameters of human health are evolving, bringing more comfort and aiming to increase people’s quality of life as well as life expectancy. However, some applications could be implemented with the aim of (i) improving diagnoses, (ii) minimizing medical errors, (iii) preventing unnecessary hospitalizations, (iv) reducing the consumption of unnecessary resources, and (v) promoting better disease management. One of the techniques that can be implemented is gamification, which may contribute to the most diverse personal factors (e.g., a feeling of integration, self-satisfaction, accomplishment, pride) and may also assist in cognitive development through knowledge acquisition.

At first glance, these “health gamification” strategies may seem to have a merely recreational character with the accumulation of points, shared achievements, and the idea of “playing” with one’s own health. However, a closer look at such applications reveals that gamification not only presents advantages in motivation and behavioral changes, but also mobilizes biopolitical calls, which invite people to manage their health, change their routines, and engage in constant self-surveillance practices. Given the above, it becomes relevant to develop applications that effectively involve the user in the long-term, engaging them in behavior changes to reduce health risks and foster disease management.

The aim of this paper is to identify the requirements for applying wearable sensors commonly found in the healthcare sector to inflammatory autoimmune diseases and propose a gamified wearable sensor for the crisis management of autoimmune diseases. The ambitions of this concept are to propose a wearable device that can sense some forms of inflammation, normally aggravated at the beginning of an autoimmune disease crisis, and inform users about preventive action behaviors (e.g., take medicine, go to the doctor), health management, and self-care, by adding motivation and behavior change elements to game design. The presentation of this concept arose from the existent gap in reviews of the literature, not only because there is very little research about the use of wearable sensors for autoimmune diseases’ patients, even if there are solutions that can be adapted to such context, but also because gamification may play a key role in the better supervision of these diseases. The present research question is: “What are the design requirements for an application to foster Inflammation Surveillance and Autoimmune Disease Management?”

A literature search was performed using the Scopus, Web of Science, PubMed, Google Scholar, PsycINFO, MEDLINE, and b-ON databases. It followed the inclusion criteria of (i) publications from the last 10 years; (ii) search results containing the paper keywords or related topics in papers’ titles, abstracts, and keywords; (iii) English and/or Portuguese publications; and (iv) open access papers. When using a combination of the terms “Wearable Sensors” OR “Sensors” OR “Wearables” AND “Gamification” OR “Gamified” AND “Autoimmune Disease” OR “Autoimmune” OR “Autoimmunity” AND “Inflammation” AND “Surveillance” AND “Self-care” OR “Self care” AND “mHealth” OR “Mobile Health” OR “Health”, no documents were found. Thus, the following review for separate topics was performed: (a) “Autoimmune Disease” OR “Autoimmune” OR “Autoimmunity” AND “Inflammation” yielded 6261 potentially eligible documents. In this search, the articles that did not have a description of the autoimmune disease or were solely related to genetics and molecular issues were excluded. Other searches performed were (b) “Wearable Sensors” OR “Sensors” OR “Wearables” AND “Surveillance” AND “Self-care” OR “Self care” AND “mHealth” OR “Mobile Health” OR “Health”, which yielded 12 potentially eligible documents; and (c) “Gamification” OR “Gamified” AND “Self-care” OR “Self care” AND “mHealth” OR “Mobile Health” OR “Health”, which yielded 54 potentially eligible documents. From these papers, the authors removed duplicated documents, which yielded a sample of 296 eligible papers. These papers enabled the gathering of some of the requirements and the concept of the wearable app iShU for inflammation surveillance and disease management, which was validated by a group of patients diagnosed with autoimmune or inflammatory diseases, health professionals, and scholars with expertise in user experience.

This paper is structured as follows: Section 2 contextualizes the connection between the inflammatory response to autoimmunity, providing a description of the most common organ-specific and systemic autoimmune diseases; Section 3 gives an overview of wearables in health monitoring within specific physiological signs; Section 4 outlines the use of gamification in mHealth with related work; Section 5 presents the prototype of a gamified app with a wearable connection to autoimmune diseases management; Section 6 discusses the presented solution for the actual market gap found in the literature search and testing; and Section 7 shares some of the study’s considerations and limitations.

## 2. Linking Inflammation to Autoimmunity

Living organisms tend to react to an aggressive stimulus (e.g., wounds or injuries) by trying to restore homeostasis (i.e., the condition of relative stability that the organism needs to perform its functions properly for the body balance), which involves the coordination of the immune, inflammatory, cardiovascular, endocrine, and nervous systems. In most situations after the organism response, healing is successful, as the host supports a suitable inflammatory response leading to the homeostasis restoration and the patient survival [6].

Inflammation is a body’s normal and natural defense reaction of the immune system to aggression that must be neutralized or eliminated. It can be generated by impurities from infectious agents (e.g., bacteria, viruses, venom, and other parasites), in addition to other factors such as heat, physical trauma, exposure to radiation, and irritating chemicals. In these situations, the body initiates the inflammatory response that aims to eliminate the cause of the injury, remove dead cells and damaged tissues, as well as start their repair.

In fact, the inflammatory response is not a single event. Instead, it is a series of events that follow a uniform path with variations in the relative intensity and duration according to the type of injury and particular aspects of the response [7]. Its events are divided into fluid (i.e., exudative) and cellular phases of circulation [8]: (i) the fluid phase reaction consists of transient vasoconstriction followed by the sustained dilation of arterioles, capillaries, and venules, during which blood flow is increased and subsequently decreased, and permeability to plasma proteins is increased; and (ii) the cellular response consists of the edema of tissue histiocytes and macrophages and the emigration of leukocytes from the vessels, preceded by the edema of vascular endothelial cells and the adhesion of leukocytes to these.

The study on inflammatory response has continuously aroused renewed interest. It happens because there are many diseases in which much of the tissue damage results from the inflammatory response itself [9,10]. Autoimmune diseases fall into this category [8], as the conditions in which the local homeostatic capacity is overcome, either by the magnitude of the aggressive stimulus or by the insufficiency of regulatory mechanisms. The inflammatory response goes beyond the limits of its microenvironment and can manifest itself in a systemic way throughout the entire organism.

Furthermore, autoimmunity may happen not only due to the dysregulation of the adaptive immune system, but also to the innate immune system [11]. The progress of autoimmune diseases can be caused by environmental factors (e.g., stress, infection, drugs, sugar and fat consumption, smoking) in genetically predisposed individuals, where the proinflammatory functions of innate immunity are stimulated and the pathological response of adaptive immunity is induced [12]. Nevertheless, it is also known that additionally to the environmental factors, the genetic, hormonal, and immunological factors are considered relevant to 50% of autoimmune diseases, whereas the remaining half percent are triggered by unknown factors [13].

The following subsections will provide an overview of autoimmune diseases, detailing those organ-specific and systemic.

### 2.1. An Overview of Autoimmune Diseases

It is important to acknowledge that people can have one or multiple autoimmune diseases, being difficult to diagnose both a single one and the coexistence of diseases. According to Marrack and colleagues [14], autoimmune diseases occur in up to 3–5% of the general population and can be classified as systemic or organ-specific (Table 1). Thus, immune responses against antigens and/or cells from various tissues produce systemic diseases, while the autoimmune response against antigens of restricted distribution to tissues or cell groups produces organ-specific diseases.

**Figure 1:**
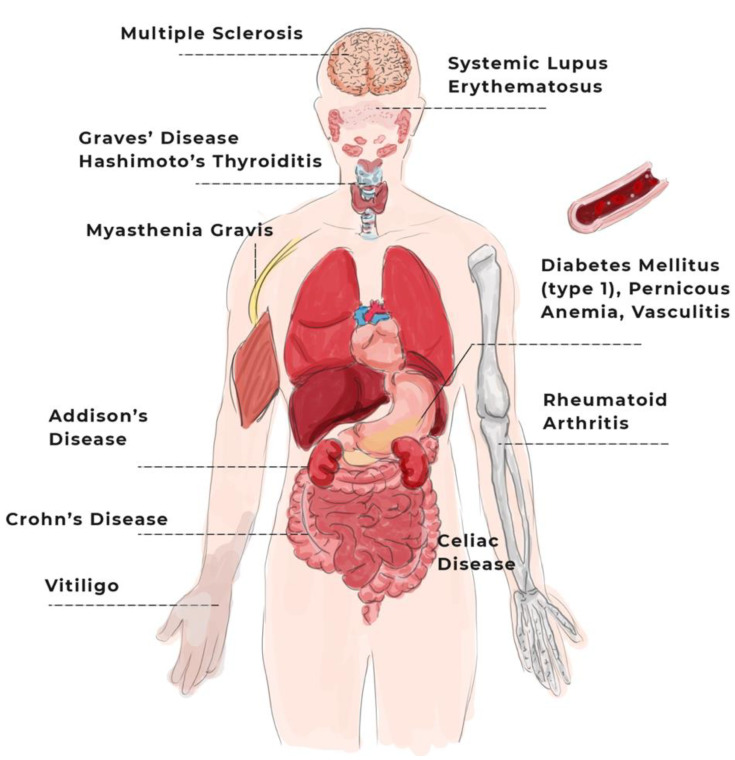
Representation of the diverse clinical manifestations of autoimmune diseases.

The clinical manifestations of autoimmunity can be diverse, varying according to the disease and the affected body part (Figure 1), from asymptomatic conditions in the presence of autoantibodies to fulminant autoimmune diseases that cause life-threatening organ damage [11], and even mortality.

Song and colleagues [15] conducted a 20-year longitudinal study to associate stress disorders with autoimmune diseases, stating that the incidence rate was 9.1 per 1000 person-years. The authors found that trauma and life stressors (e.g., grief; financial, family, health problems; a desire for social approval; pressure at work; life changes; lack of time for leisure; disasters; violence) may trigger an array of physiologic alternations, which tend to influence the immune function and develop or aggravate the autoimmune disease [15].

**Table 1:** Examples of organ-specific and systemic autoimmune diseases [14,16,17,18].

Autoimmune Disease	Organ
Organ-specific	
Addison’s Disease	Adrenal
Ankylosing Spondylitis	Spinal cord
Bullous Pemphigoid	Skin
Celiac Disease	Small bowel
Chronic Active Hepatitis	Liver
Crohn’s Disease	Bowel
Diabetes Mellitus (Type 1)	Pancreas β cells
Gastritis	Stomach
Graves’ Disease	Thyroid
Goodpasture’s Syndrome	Lungs and Kidney
Hashimoto’s Thyroiditis	Thyroid
Myasthenia Gravis	Muscle
Multiple Sclerosis	Brain and Spinal cord
Pemphigus	Skin and Mucosa
Pernicious Anemia	Stomach
Primary Biliary Cirrhosis	Liver bile ducts
Uveitis	Eyes
Vasculitis	Blood vessels
Vitiligo	Skin
Systemic	
Behçet	Blood vessels, Eyes, Skin, Genitalia, and Muscles
Cogan’s Syndrome	Eyes, Ears, Blood vessels, Lungs, Heart, Joints, and Muscles
Dermatomyositis	Skin, Skeletal muscle, Lungs, Heart, and Joints
Rheumatoid Arthritis	Joints, Lungs, and Heart
Sjögren’s Syndrome	Lacrimal and salivary glands, Lungs, Kidney, and Nervous system
Scleroderma	Blood vessels, Muscles, and Internal organs
Systemic Lupus Erythematosus	Skin, Joints, Kidney, Brain, Lungs, and Heart

Even if these diseases attack different organs and manifest in a variety of body parts in distinctive ways, triggers, such as stress, may arouse similar symptoms. The most common, predominant, and holistic (early) symptoms are inflammation and overall pain, fatigue, sore muscles, swelling, redness, low-grade fever, numbness in hands and feet (generally associated with Raynaud’s syndrome), and skin rashes [19].

Briefly and alphabetically, the following section presents a description of the autoimmune diseases in Table 1, as well as the common symptoms in the active phase of the disease and/or during crises. The specification of these diseases is relevant in order to understand the context in which wearable sensors for inflammation surveillance and autoimmune disease management can be used.

#### 2.1.1. Organ-Specific

In this subsection, different organ-specific diseases and their main symptoms are presented. These fall into the following headings: Addison’s disease, Ankylosing Spondylitis, Bullous Pemphigoid, Celiac Disease, Chronic Active Hepatitis, Crohn’s Disease, Diabetes Mellitus, Gastritis, Graves’ Disease, Goodpasture’s Syndrome, Hashimoto’s Thyroiditis, Myasthenia Gravis, Multiple Sclerosis, Pemphigus, Pernicious Anemia, Primary Biliary Cirrhosis, Uveitis, Vasculitis, and Vitiligo.

Addison’s disease is a fallout from the autoimmune destruction of the adrenal cortex and the injury of the adrenal glands [20] and is associated with a high frequency of organ-specific endocrine and nonendocrine manifestations [21]. The main symptoms occur gradually and include hyperpigmentation, exhaustion, general feebleness, weight loss, nausea, vomiting, abdominal pain, wooziness, tachycardia, and/or postural hypotension [20].

Ankylosing Spondylitis is an incapacitating inflammatory disease of the spine [22], being a severe form of spondyloarthropathies that affects not only the spine joints, but also sacroiliac joints, and their adjacent soft tissues (i.e., tendons and ligaments) [23]. The predominant symptom is inflammatory low back pain and stiffness, but there may also be generalized joint pain, decreased chest expansion from diffuse costovertebral involvement, low-grade fever, fatigue, anorexia, weight loss, and anemia [24].

Bullous Pemphigoid is a common skin autoimmune disease that mainly affects older adults and is characterized by indiscriminate crops of tense and pruritic cutaneous blisters [25]. Symptoms are based on skin inflammation, itchiness, eczematous, urticarial, and papular and/or nodular lesions [26].

Celiac Disease is a multifactorial autoimmune disease activated by the consumption of gluten in genetically susceptible individuals [27]. It is portrayed by an inflammatory injury to the small bowel with abdominal distention, chronic diarrhea, micronutrient deficiencies, fatigue, migraine, and irritable bowel syndrome [28].

Chronic Active Hepatitis is a chronic inflammatory liver disease caused by interface hepatitis and lymphocytic infiltration [29]. Some of the symptoms are fatigue, abdominal pain, joints stiffness, nausea, anorexia, and malaise; however, this disease may lead to acute liver failure that requires liver transplantation [30].

Crohn’s Disease is a chronic inflammation of the gastrointestinal tract that occurs due to genetic susceptibility, environmental factors, and altered intestinal microbiota [31]. Common signs include chronic repetitive ulceration of the intestines, diarrhea, obstruction, rectal bleeding, abdominal pain, anemia, fatigue, and weight loss [32].

Diabetes Mellitus, also named autoimmune diabetes or Type 1 diabetes mellitus, is a chronic immune-mediated illness depicted by insulin defects due to pancreatic islet beta-cell damage with growing blood glucose levels [33]. The main symptoms are weakness, weight loss, chronic or intermittent ingestion of large volumes of water (i.e., polydipsia), the passage of large volumes of dilute urine (i.e., polyuria), and vulnerability to infections [34].

Gastritis is a stomach immune-mediated disorder characterized by the destruction of gastric parietal cells, leading to an intrinsic factor (i.e., glycoprotein) deficiency and low acid output [35]. The disease is expressed by fatigue, weakness, pale appearance, anemia, and subacute combined degeneration [36].

Graves’ Disease is a hyperthyroidism immune illness, defined by a diffuse hyper-functional goiter [37]. There is a prevalence of weakness, polydipsia, visible goiters, weight loss, arrhythmia, tachycardia, intolerance to high temperatures (i.e., thermophobia), palpitations, and digestive and sleep disorders as symptoms [38].

Goodpasture’s Syndrome is a pulmonary and renal autoimmune disease that usually presents acute kidney failure and bleeding in the lungs, which may lead to death [39]. Individuals with this disease may have blood-spitting (i.e., hemoptysis), blood in the urine (i.e., hematuria), chest pain, hypertension, malaise, and weight loss [40].

Hashimoto’s Thyroiditis is the most dominant autoimmune thyroid disorder, consisting of gradual damage to the thyroid gland (i.e., hypothyroidism) [41]. A variety of symptoms, such as muscle weakness, fatigue, and depression, may be found [42].

Myasthenia Gravis is a muscle-specific autoimmune disease triggered by the malfunction of neuromuscular transmission [43]. Some of the symptoms described in the literature are muscle weakness and fatigue, respiratory limitation, double vision (i.e., diplopia), swallowing difficulties (i.e., dysphagia), upper eyelid droops (i.e., ptosis), motor speech disorder (i.e., flaccid dysarthria), and facial and jaw paralysis [44].

Multiple Sclerosis is characterized by the early inflammation, due to autoimmunity, that causes neurodegeneration of the central nervous system [45]. Some indicators of this autoimmune disease are fatigue, general pain, facial weakness (e.g., electric shocks, hemifacial spams, rapid contractions), lack of motor coordination (i.e., ataxia), sensory loss, swelling of the optic nerve (i.e., optic neuritis), diplopia, brain fog (i.e., cognitive deterioration), heat sensitivity, vertigo, and sexual and bladder dysfunction [46].

Pemphigus is a heterogeneous group of autoimmune bullous diseases that disturb the skin and mucosa [47]. Individuals with pemphigus tend to present flaccid skin vesicles, redness (i.e., erythematous), erosions, seborrhea, bacterial/viral superinfections, hypo- and hyperpigmentation, and also oral lesions similar to aphthae and herpes [48].

Pernicious Anemia is megaloblastic anemia (i.e., deficiency of vitamin B12) caused by the lack of intrinsic factors and characterized by the inflammation of the stomach [49]. Even if this disease is gastric, symptoms may also appear at intestinal, hematological, neurological, cardiac, and urinary levels. Some of the clinical findings are low-grade fever, lethargy, fatigue, headache, faintness, tachycardia, abdominal bloating, weight loss, diarrhea, ataxia, paresthesia, palpitations, edema, urinary retention, memory loss, and psychosis [49,50].

Primary Biliary Cirrhosis is a chronic liver disease with a decrease in bile flow (i.e., cholestasis), characterized by damage to small intrahepatic bile ducts, which leads to biliary fibrosis, cirrhosis, and liver failure [51]. Fatigue, pain and discomfort, itchy skin (i.e., prutitus), the accumulation of fluid in the abdomen (i.e., ascites), hepatic encephalopathy, esophageal variceal bleeding, the yellowing of the skin/sclera (i.e., jaundice/icterus), sleep deprivation, depression, and weight loss may occur [52].

Uveitis is an ophthalmological autoimmune disease characterized by the inflammation of the eye [53]. The main symptoms are eye redness, swelling pain, and blurred vision [54].

Vasculitis is a generalized term for the diseases’ characterization of the inflammation of blood vessels [55]. Common symptoms are fever, loss of appetite, weight loss, fatigue, myalgias, stiffness [56], rash, numbness, and weakness.

Vitiligo is a depigmenting skin condition due to the destruction of epidermal melanocytes [57]. The predominant symptoms are the lack of pigmentation in patches of skin, retina, hair, and mucous membranes (i.e., white spots that tend to enlarge over time), and depression [58].

Having mentioned the organ-specific diseases, the next subsection will provide an overview of the systemic diseases.

#### 2.1.2. Systemic

As mentioned in the section ‘An Overview of Autoimmune Diseases’, systemic diseases are relative to immune responses against antigens and/or cells from various tissues. Some examples include Behçet, Cogan’s Syndrome, Dermatomyositis, Rheumatoid Arthritis, Sjögren’s Syndrome, Scleroderma, and Systemic Lupus Erythematosus.

Behçet disease (or syndrome) is a complex, multisystemic inflammatory vasculitis characterized by a relapsing and remitting course. It can involve, one by one or simultaneously, the skin, mucosa, joints, blood vessels, eyes, and nervous and gastrointestinal systems [59]. Some of the distinctive symptoms are oral and genital aphthae and ulcers, ocular crisis, and vascular inflammation; however, acne, arthritis, stomach, and intestine bleeding and abnormalities in the nervous system may arise [60].

Cogan’s Syndrome is a rare autoimmune disorder characterized, mainly, by ocular (i.e., interstitial keratitis) and audiovestibular symptoms; however, it also presents a multisystemic involvement that may include joint and muscle pain; the inflammation of the blood vessels; and/or gastrointestinal, heart, and lungs problems [17,61].

Dermatomyositis is an idiopathic inflammatory myopathy characterized by diverse skin injuries [62]. Symptoms may be the cutaneous disease alone, simultaneous muscle malady, or have extracutaneous manifestations, such as lung and heart disorder [63].

Rheumatoid Arthritis is a systemic autoimmune disease characterized by the inflammation of the joints that may cause their destruction and the alteration of the bones’ pattern and extra-articular manifestations at pulmonary and cardiovascular levels [64]. The common symptoms are joint pain (i.e., arthralgia), swelling, stiffness, redness, warmth and burning sensations, weakness, loss of motor coordination, fatigue, sleep deprivation, depression, cramps, numbness, atypical skin sensation, weight loss, transient nodules [65], fluid on the lungs (i.e., pleural effusion), dyspnea, cough, fever, chest pain [66], and hypertension [67].

Sjögren’s Syndrome is portrayed by the occurrence of periductal lymphocytic infiltrations in salivary and lachrymal glands resulting in oral and ocular dryness [68]. Typical signs are dry eyes and mouth (i.e., xerophthalmia and xerostomia), dry skin, fatigue, pain, dysphagia, distorted taste sensation (i.e., dysgeusia), burning impression, dental caries, periodontal disease, photosensitivity, redness, itching, hoarseness, nonproductive cough, painful intercourse (i.e., dyspareunia), sleep disorder, anxiety and depressive symptoms, stiffness, pain in muscles (i.e., myalgia), arthralgia, weakness, pallor, cutaneous vasculitis, and paralysis [69].

Scleroderma is a multisystem autoimmune disease depicted by inflammation, autoantibodies, vasculopathy, and the fibrosis of the skin and internal organs [70]. It is characterized by Raynaud’s syndrome, skin contraction and thickening, gastrointestinal dysmotility, fatigue, weakness, difficulties in breathing, arthralgia, and physical activity intolerance [71].

Systemic Lupus Erythematosus is one of the most heterogeneous systemic autoimmune diseases that may affect organs and tissue [72]. Signs include fever, arthralgia, fatigue, pain, depression, Raynaud’s syndrome, photosensitivity, oral ulcers, malar rush (i.e., butterfly redness effect on the face), scaly, rosacea, hair loss (i.e., alopecia), joints swelling (i.e., synovitis), pleural effusion, seizures, and psychosis [73].

Considering the common symptoms faced in organ-specific and systemic autoimmune diseases, it is necessary to be aware and detect them in earlier phases of the inflammatory process to prevent further health decay. Thus, the following section provides wearable sensors for identification of physiological signs in health monitoring.

## 3. Wearable Sensors in Health Monitoring

Ubiquitous computing is a concept based on software engineering and computer science that is concerned with incorporating computational capability into any instrument or device invisibly in daily life. Wearables consist of technological devices worn by a user under, over, or even incorporated into clothing accessories, being usually small in size and easily adapted to a particular task.

Wearable devices allow various data collections, enabling an increasingly targeted and personalized use that could assist with self-diagnosis and behavior change interventions [74]. It implements an alternative form of communication between humans and computers, facilitated by using sensors. Such technology can be used to obtain physiological data from users, and this type of information may constitute knowledge applicable to preventive medicine procedures. In fact, long-term remote monitoring has the potential to enable early diagnoses and interventions, leading to a reduction in frequent visits to hospitals and clinics [75].

The use of mHealth technology (i.e., mobile communication technologies for access to health services and information) to reduce the risk of the development of certain diseases is promising. However, its integration into routine clinical care and population health management has been a major challenge [76]. Despite visible barriers such as the lack of data integration in medical records, wearable health devices are making progress in terms of ease of use, reliability, and automation. The advancement and evolution of mHealth solutions also seems to rely on the definition of clinical guidelines that help to standardize data capture and integration [76].

According to Metcalf and colleagues [77], health and human fitness are areas in which wearable devices have the greatest data collection capability. Compared to smartphones, these devices are likely to be more suited to self-monitoring physical activity, despite a notable lack of the potential user’s engagement. Still, doctors could suggest using these devices for patients to monitor health conditions (e.g., hypertension and stress).

Motti and colleagues [78] discuss the requirements, challenges, and opportunities for potential wearable applications aimed at general health care, particularly behavioral changes, presenting a brief analysis of the state of the art. The authors demonstrate various applications using accelerometers to detect the progression of chronic diseases and the use of other sensors, such as skin conductance, to identify stress. Some requirements for designing wearable devices are highlighted [78]: (i) continuous data collection; (ii) discreet and ergonomic design to maintain contact with the body; (iii) simple access to data; (iv) easy interaction; (v) reliability; (vi) intuitive interface; (vii) data privacy; and (viii) custom interfaces which consider the user experience.

The following subsection gives an overview of the most common types of physiological detection in wearable sensors.

### 3.1. Physiological Detection

**Figure 2:**
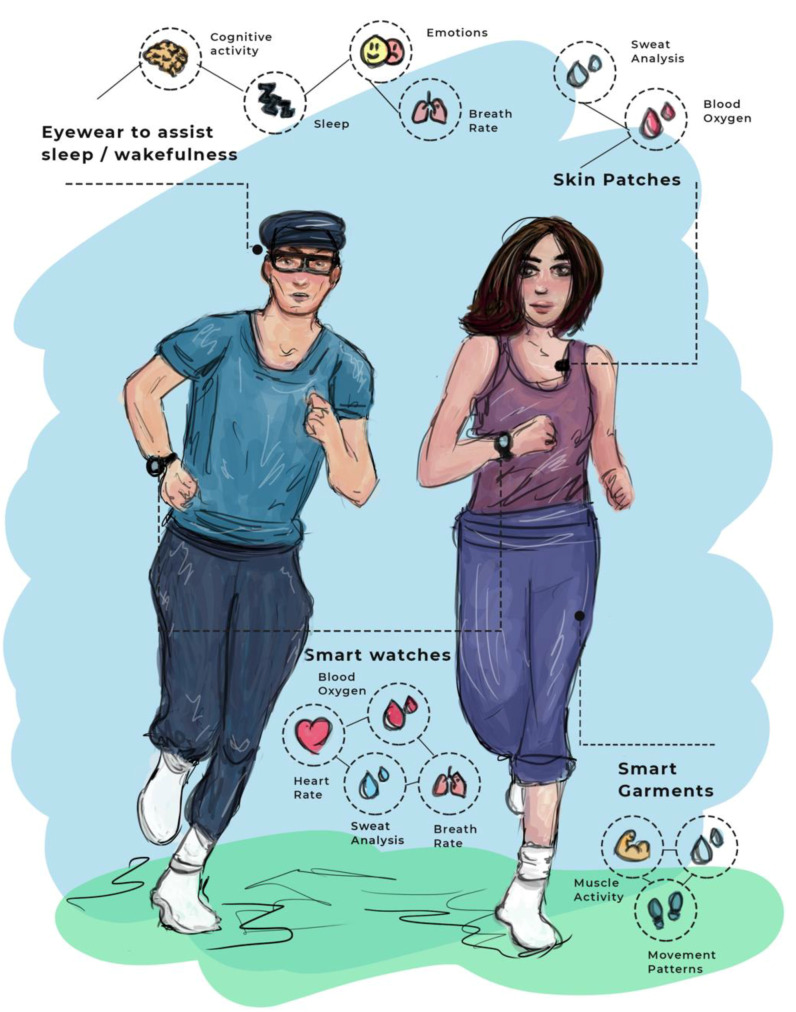
Representation of wearable sensors and detected physiological signs.

Portable biosensors enable the measurement of various physiological health-related variables, as demonstrated in Figure 2. According to Li and colleagues [79], wearable devices are capable of measuring daily physical activity, as well as measuring parameters, such as heart rate and skin temperature, although their use serves to (i) accurately record physiological measurements in individuals at real-time and/or at high frequency; (ii) quantify daily patterns and reveal interesting physiological responses to different circadian and environmental cycles; (iii) identify personalized benchmarks and differences between individuals; (iv) detect disparities in health status between individuals (e.g., people with diabetes versus people without diabetes); and (v) detect inflammatory responses and aid in medical diagnosis at an early stage of disease development, potentially impacting medical care.

Several studies review wearable sensors in stress and pain detection [80,81,82], electrical brain activity [83], skin hardness [84], hypoglycemic events [85,86,87,88], and inflammation [89]. The useful physiological signs described in the mentioned studies are heart activity (i.e., ECG—Electrocardiogram) and blood volume pulse (BVP), brain activity (i.e., EEG—Electroencephalogram), muscle and neural activity (i.e., EMG—Electromyography), electrodermal activity (EDA), body temperature, and respiratory activity, which are organized in Table 2 with the attributed commercialized and divulgated wearable sensors. Moreover, other wearables, such as body bands/belts and smartwatches (e.g., Garmin, Wahoo, Apple Watch, miBand, Samsung Watch, Huawei Watch)—as well the sensors in the previous authors’ studies—are also able to detect physiological signs (e.g., heart rate, sleep activity) that help in health monitoring.

The following subsections describe the health monitoring in each category of physiological signs portrayed in Table 2 and Figure 2.

#### 3.1.1. Heart Activity and Blood Volume Pulse

Cardiovascular diseases are one of the main reasons for death in humankind; therefore, information on heart rate is vital in order to diagnose and prevent the exacerbation of critical states. It is possible to measure the electrical activity of the heart (e.g., heartbeat, rhythm, pulse pressure) with an ECG, since it enables the detection of the contraction and relaxation movements of the myocardium or valves and the identification of ischemia (i.e., restriction of blood flow to the heart tissue) [90].

Nowadays, wearable devices—such as wrist bands and smartwatches—have biological sensors; thus, their complexity has not only the potential to track health, wellness, and fitness but also the potential to diagnose cardiovascular diseases. This can be prescribed by a health care provider and influence therapeutic decisions [91].

According to Singhal and Cowie [92], most of the wearable sensors that monitor heart activity utilize photoplethysmography for this purpose (i.e., a non-invasive, simple, and low-cost optical technique, by light transmission or reflection, that can be used to detect blood volume changes in the microvascular bed of tissue). It uses a light-emitting diode (LED) to shine on the skin, specifically a capillary bed, detecting a pulse by measuring the difference in the quantity of light reflected the sensor, and thus monitoring for pulsatile changes in light absorption [93]. Such trackers can map the cardiovascular activity, monitoring abnormalities in the heart function, and may even prevent heart failure and provide further diagnosis.

#### 3.1.2. Brain Activity

The brain plays a fundamental part in controlling various biological and physiological processes, aside from changes in our body. Thus, supervising patients with cerebrovascular and paroxysmal pathologies through EEG has been considered an important tool both for the diagnosis and classification, as well as for the treatment of these conditions. EEG measurements involve an array of electrodes placed on patients’ heads that record event-related potentials in the event of brain stimulation. However, like ECG analysis, measurements are filtered across chest straps and smartwatches that can be analyzed via power spectrum relationships [94]. Wearables that contain sensors to measure EEG signals that use electrical and power potentials can monitor and maintain brain health, giving neurofeedback about the way people think, feel, and sleep [83].

Nevertheless, EEG is a technique that detects general electrical activity in the outermost groups of brain cells, being unable to pick up signals generated in their internal structures. Thus, the identification of drowsiness by means of EEG electrodes is only possible because the intralaminar centers of the thalamus contain thalamocortical neurons that send projections throughout the cortex. In this way, the ongoing tug of war between sleep and arousal that occurs in the brainstem controls the mental state, modulating the rhythmicity of these thalamocortical interactions [95]. The activation of thalamocortical neurons causes them to release excitatory amino acids, such as aspartate and glutamate, thus contributing to the excitation of the cortex, which keeps the individual awake. During the waking state, these neurons generate isolated action potentials at regular intervals, but as the individual falls asleep, these neurons begin to fire in bursts, causing the cortex to display the synchronized EEG pattern typical of sleep [95].

The peak cluster activity of cortical surface neurons accumulates field potentials that are detected by electrodes on the scalp as the characteristic waves and complexes that guide electroencephalographs in EEG interpretation.

#### 3.1.3. Muscle and Neural Activity

EMG is a technique for evaluating the electrical activity produced by skeletal muscles when their cells are electrically (e.g., by factors external to the body) or neurologically (e.g., by the body’s own activities—voluntary or not) activated. The potential difference between one part of a muscle relative to another is measured overtime during the execution of some movement or at rest [96]. The signals generated by electromyography have several clinical and biomedical applications. On the one hand, some examples of clinical applications are the evaluation of signs for (i) detection of anomalies, (ii) causes of muscle pain, (iii) atrophies, (iv) arthralgia, (v) osteoarthritis, and (vi) rheumatism. On the other hand, biomedical applications can be used for human–machine interaction and prosthesis construction.

According to Raez and colleagues [97], there are two forms of EMG capture: (i) superficial and (ii) intramuscular. Superficial EMG uses electrodes connected to the part of the skin that is above the muscle to be evaluated. Despite being a less invasive approach for the patient, it is more limited in relation to the intramuscular form due to the fact that the contact of the electrodes is restricted to the superficial muscles and also because it is influenced by the depth of the subcutaneous tissue at the recording site, in addition to not being very accurate in discriminating between discharges from muscles adjacent to the assessed muscle. Intramuscular EMG, on the other hand, consists of the use of needles to directly access the muscles to be evaluated, with much greater precision, although with a much more invasive and delicate application.

#### 3.1.4. Electrodermal Activity (EDA)

The skin is a selective barrier that prevents foreign bodies from entering the body and facilitates the specific passage of materials from the bloodstream to the outside of the body. It also acts as a regulator of body temperature, a function primarily performed by vasoconstriction and vasodilation, but also by the variation in sweat production, which is the most important aspect for the study of EDA. EDA, also known as galvanic skin response, refers to the variation of the skin electrical conductance response, skin potential, skin conductance level, and skin potential response [98].

Due to its usefulness as an indicator of an individual’s stress, the electrodermal signal is often used in psychological studies to measure anxiety [99]. This signal, rather than the EEG, is used to assess an individual’s neurological status, as electrodermal activity is more susceptible to emotional changes than cardiac activity [100]. The EDA signal is used simultaneously with blood pressure, heart rate, and respiratory rate in polygraphs as lie detectors, since they are autonomic responses of the nervous system and are difficult to consciously alter [101]. The electrodermal signal is also used in conjunction with heart rate in video game and virtual reality development [102] and, similarly to heart rate, electrodermal activity can translate the level of enthusiasm, frustration, boredom, and game enjoyment. In this way, collecting and analyzing these signals allow producers to adapt their video game to the appropriate level of desired emotion.

Zhang and colleagues [103] created a flexible electrochemical biosensor based on silver nanowires by molecular imprinting to monitor lactate in perspiration during physical exercise. This biosensor, which is a polydimethylsiloxane flow cell, is implanted in a flexible substrate by a screen-printing process. Thus, the lactate return current is captured with measurement in the epidermis. Promphet and colleagues [104] created a wearable colorimetric sensor that is based on altered cotton threads to noninvasively and synchronously detect glucose and urea excreted in human sweat. Of the traditional analytical techniques, colorimetry was chosen due to its practicality for self-monitoring, simplicity, easy interpretation with the naked eye, and self-detectability, not requiring blood to perform the monitoring [104].

Furthermore, sweat-based wearables are noninvasive sensors that not only can measure glucose levels essential in diabetes diagnosis and monitoring [104,105], but also prevent kidney failure [104].

#### 3.1.5. Body Temperature

Body temperature corresponds to the average temperature of the human body. On average, it is around 36 to 37 °C (97 to 99 °F), with variations depending on age, gender, emotions, activities, and the time of day. Several diseases, infections, and deteriorating conditions are identified by changes in body temperature; thus, measuring body temperature is considered an important parameter in medicine [106].

On the one hand, hyperthermia (i.e., the elevation of body temperature above the normal range) can occur in patients not only with infections, brain abnormalities, toxic substances that affect thermoregulators, bacterial diseases, brain tumors, and environmental conditions, which can cause great damage to the brain and other organs of the human body, but also with inflammatory and autoimmune diseases. On the other hand, hypothermia (i.e., a decrease in body temperature to values below 35 °C), which can be caused by severe and sudden bleeding disorders, diabetic coma, artificial freezing, shock, and the terminal stages of many diseases.

According to Kyriacou [107], several sensor devices have been developed to detect temperature through specific physical changes, including resistive temperature detectors (RTD), thermally sensitive resistors (i.e., thermistors), mercury thermometers, infrared sensors, thermocouple sensors, field-effect transistors, optical sensors, and silicon sensors, as well as luminescent materials.

#### 3.1.6. Heart Activity and Blood Volume Pulse

The respiratory process is directly related to the maintenance of life. Essentially, it consists of the absorption of oxygen by the body and the elimination of carbon dioxide, from cellular oxidations (i.e., exchange of gases between the atmosphere, lungs, blood, and cells). Techniques for monitoring respiratory activity can be divided into direct and indirect techniques [108]: some of the direct techniques are spirometry, pneumography, and resistive transducers; in indirect techniques, the physiological magnitude of respiration is taken from the measurement of other physiological phenomena, such as electrocardiography or photoplethysmography.

Respiratory failure can be life-threatening; thus, abnormalities in respiratory rate, depth, and pattern are a strong predictor of acute events (e.g., cardiac arrest), to characterize pulmonary diseases (e.g., chronic obstructive pulmonary disease, pneumonia, asthma) [109], or of identifying other breathing disorders (e.g., sleep apnea).

Biomedical sensors accurately monitor respiration rate through sound, airflow, and chest movement [110]: (i) a thermistor is typically used for detection, as exhaled air is usually warmer than inhaled air, and the cyclic change in temperature can be transduced and correlated with a rate of breathing; (ii) a commonly used biofeedback wearable is the RSA (Respiratory Sinus Arrhythmia), which is able to measure the variation in respiratory rate through a digital infrared sensor; (iii) other technologies measure lung activity through a pressure sensor (i.e., the increase in pressure is registered by the sensor when the individual exhales air); and (iv) capnography-monitored breathing, especially in the context of anesthesia, emergency medicine, intensive care units, and sleep medicine. In addition, sound-based respiration rate detection (v) using acoustic sensors (e.g., microphones) has been suggested as an alternative.

Since sensors are able to provide a great amount of biofeedback, it is important not only to connect them with apps that can communicate such information, but also to keep the user engaged in the use of sensors for health monitoring. Thus, the following section shows the importance of gamification in mHealth for behavior change.

## 4. Gamification in mHealth

The mHealth apps allow users to interact with their smartphones to monitor and control their health status; however, one of the greatest challenges in the mobile applications market is to maintain user engagement with the application on a long-term or recurring basis, without the loss of interest. Engagement is a consequence of the depth of participation that the user is able to achieve in relation to each function available in the application, also considering aesthetic features, usability, intuitive visual structure, ability to be present, and involvement in a given experience during the execution of tasks [111]. One of the most used techniques to motivate and engage the user is gamification, targeting users’ behavior changes, social interactions, and task/goal achievement.

Gamification is defined as the use of game design elements, in a nongame context, with the purpose of motivating and involving users in different activities [112]. According to Ryan and Deci [113], there are two types of motivation: (i) extrinsic and (ii) intrinsic. Extrinsic motivation is related to the environment or situations (e.g., rewards, bonuses for achieving a certain goal, or to avoid punishment), and needs to be stimulated as it tends to be fickle because it depends on external factors. Intrinsic motivation, on the other hand, originates from internal factors (e.g., interests and tastes), and tends to be constant, as it depends solely on the individual and not on external factors.

In the health sector, the lack of engagement for the continuity of treatment is a constant concern for specialists and doctors, who have been adopting technology as a motivational factor. Although mHealth applications bring benefits and provide information capable of assisting in treatment, a study by Ribeiro and colleagues [114] draws attention to the fact that the vast majority of users do not act in accordance with the proposed recommendations, highlighting the main causes of abandonment reported by users (e.g., abandoning use after reaching or completing a goal and the lack of available resources).

According to Schmidt-Kraepelin and colleagues’ taxonomical classification [115], gamification approaches in mHealth apps consists of: (i) concept-to-user communication, in which the messages can be delivered directly or indirectly (i.e., mediated); (ii) users’ identity, based on their representation, which can consist of customized avatars or be self-selected; (iii) rewards, which can be internal (i.e., virtual and with no real-world value) and external (i.e., virtual with real-world value); (iv) competition, divided between direct (i.e., competition against other users on the same task) and indirect (i.e., the comparison of performance with other users); (v) target groups, which can be used for patients, healthy individuals, and health professionals; (vi) collaboration, which may be cooperative (i.e., users help each other in the accomplishment of a task) and supportive (i.e., users may be motivated to accomplish a task without direct help from others); (vii) goal setting, which may allow for self-set (i.e., users define their goals) or externally set (i.e., set goals defined by external stakeholders); (viii) narrative, segmented into episodical (i.e., the progress of the user may be reset partially or fully) or continuous; (ix) reinforcement, either positive (i.e., with emphasis on the users’ success) or positive–negative (i.e., referral to failures or unhealthy status); (x) persuasive intent, which approaches compliance change (i.e., encourage users to be obedient to specific rules and guidelines), behavior change (i.e., motivate healthy behaviors in specific contexts without strict rules), and attitude change (i.e., influence users attitude towards a goal); (xi) level of integration, which may be inherent (i.e., health-related activities partially or fully embedded in the gamified mHealth) or independent (i.e., health-related activities that can be easily performed without the gamified mHealth); and (xii) user advancement, which can be progressive (i.e., the unlocking of resources) and presentation only (i.e., only the progress is represented).

The next subsection examines published work on the use of gamification elements in mHealth apps.

### Related Work

According to Sardi and colleagues [116], the highest emphasis in gamification health is on physical activity, dieting, physical and mental rehabilitation, and chronic disease management (e.g., cardiovascular diseases and diabetes). Apps such as Nike+, Fitbit, MySugr, and MyNetDiary are examples of gamified mHealth, the first two being linked to exercise and heart rate monitoring, whereas the last two are for diabetes supervision. Thus, in this subsection, research that is more directly related to this matter is presented.

Octopus is the work presented by Paim and Barbosa [117], which is a gamification model for the assistance in the ubiquitous care of chronic diseases (e.g., diabetes mellitus, obesity, and depression) and seeks to encourage the use of context-sensitive resources by promoting changes in user behavior. It has the central idea of making users apply medical measurement tools (e.g., blood glucose, weight), earn rewards for completing tasks, and suggests, for example, nearby restaurants specializing in sugar-free foods, low-fat foods, points for physical activities, and places to use glucometer sensors. The following features can be highlighted: (i) a vital signs viewer that allows the simple and quick identification of the indicator level, (ii) weight and activity charts, (iii) support for the definition of objectives, (iv) the visualization of progress and objectives achieved, (v) scoring, (vi) alerts, and (vii) notification via SMS if any vital sign is different from what was expected.

Wahle and colleagues [118] presented MOSS, a context-sensitive system to detect depressive symptoms and provide interventions for people using the system. The system is based on the patient’s daily behavior mapped through common sensors available on a smartphone. Moreover, MOSS uses the GPS sensors, accelerometer, and smartphone usage duration to monitor patients’ behavior. The following features are present: (i) activity monitoring, which encourages physical activity that is monitored through the detection of walking time; (ii) quizzes, asking questions for the patient to answer through multiple choice answers; (iii) checkboxes, when presenting a statement to the patient and the patient check a box if the statement is true; (iv) a button to encourage the user to perform physical exercises by pressing a button that releases a countdown timer; (v) a mirror, for the psychological and therapeutic effect through the front camera, encouraging patients to look at themselves; (vi) audio with exercise instructions for patients to perform; (vii) multi-text, with educational and instructional texts; and (viii) a stopwatch, where the time starts counting for the user to perform some intervention until a signal is given.

In the work of Goh and colleagues [119], the use of gamification to encourage the performance of the heel lift exercise in older adults is discussed. This exercise, which involves standing on your feet and elevating your heels, is a fundamental exercise in physical therapy and rehabilitation. The work proposes a gamified application, where users interact with an avatar that jumps platforms from jump to jump. The jump is performed in the application as soon as the exercise is performed. For this, the smartphone application on the user is connected by Bluetooth to specific hardware that contains pressure sensors.

Sousa and Alves [120] developed a gamified app for the treatment of children with faintness, entitled Dizziness Kids App. The optokinetic training is performed through the virtualization of the Barany Drum (i.e., cylinder composed of white and black stripes) placed in front of the patient to rotate, clockwise and counterclockwise, while the patient follows the stripes with repetitive movements, performing an optokinetic training. This occurs because the vestibular system, which is responsible for the individual’s balance, adapts as it reacts to these optokinetic stimuli, which leads to a decrease in balance oscillation. Regarding gamification: (i) The stimulation time is selected according to the child’s need and age through a mascot. This stimulus aims to enable the child’s engagement and entertainment to stay in the environment and carry out the proposed training daily, being rewarded with points; (ii) the reward system was defined based on user interaction, which increases the score every time the pet is activated. In the short term, the user has the possibility to buy items in the store or to personalize the mascot upon completing 100 points; and (iii) the feedback system is mediated and visualized by the child, as it also transmits sounds and numbers that inform of the successes and errors (although there is the possibility of silencing the sound during training).

Furthermore, studies directly linked to autoimmune diseases can also be found, with an emphasis on organ-specific autoimmune diseases, such as diabetes mellitus and multiple sclerosis. An example of the self-care promotion of diabetes mellitus is DM Agendinha (i.e., Diabetes Mellitus Agenda), an application for youngsters [121]. It is aimed at controlling blood glucose in children and adolescents; it has a friendly interface and allows the recording of blood glucose, providing a graph with the history, medications, meals, and indications of sports practices, prevention, and coping with complications. In addition, it has a list of songs for relaxation to stress-fighting.

In the same vein, MyDiabetes is a mHealth app targeted at the tracking and monitoring of type I diabetes that added gamification elements to increase the patients’ participation and engagement [122]. The app was developed based on the literature review and medical expert’s opinions, incorporating (i) the daily information about the users’ daily management of the disease (e.g., glycemic values); (ii) feedback based on color values (e.g., green for good values, yellow requires caution, and red demands the users’ attention); (iii) a social community with anonymous leaderboard; (iv) widget for an instant view of the last records and evaluation of the tendencies of carbohydrates, insulin, and glycemia results; (v) a notification with motivating feedback; (vi) a reward system based on badges; and (vii) progress with association to the difficulty levels.

Another example is the H2Overload^®^ application, developed by the National Kidney Foundation (NKF), which allows the managing of water intake and also the use of product barcodes to obtain Nutritional information [123]. The app is recommended for use by patients with chronic kidney and end-stage renal diseases (e.g., Goodpasture’s Syndrome), covering various aspects of self-management, such as knowledge about the pathology, medication management, modification of life habits, establishing positive attitudes towards well-being and health mental health, relationship with health professionals, and active participation in health, recognizing and identifying symptoms [124].

Additionally, Giunti and colleagues [125] developed More Stamina, a user-centered gamified mHealth for multiple sclerosis. It is a chore organization tool that helps patients save energy to minimize the impact of their symptoms of fatigue. The app relies on (i) a reward system with stamina credits (i.e., energy as virtual currency or points), (ii) positive feedback, (iii) reminders, (iv) progress, (v) challenges, and (vi) social media connection. According to the authors, the app could be used for other diseases that experience fatigue as a symptom [125].

Taking into consideration the existing relevant work, the authors propose the use of gamified systems in inflammation surveillance and autoimmune disease management in the following section.

## 5. Gamified Inflammation Surveillance of Autoimmune Diseases: A Proposal

Research and development solutions in the context of autoimmune diseases, gamification, and wearable sensors are still lacking. It is possible to find gamified mHealth for a few organ-specific autoimmune diseases, such as diabetes mellitus, celiac disease, and multiple sclerosis (e.g., [126]), as well as an activity tracker for the promotion of physical activity in systemic arthritis rheumatoid (e.g., [127]) or support in systemic lupus erythematosus (e.g., [128]). However, most of these are only able to track and monitor patient-reported symptoms, providing educational content or forming a support group network [128] without integrating a gamified strategy or offering connectivity with wearable sensors or incorporating methods of passive data collection.

Given the literature search on autoimmune diseases’ common symptoms that are usually linked to inflammation exacerbation, it is possible to highlight fever, redness, swelling/edema, a sensation of heat, fatigue, numbness, physical pain, and depression. Cross-referencing with the literature on wearable sensors, such symptoms may be identified in Table 2 relative to mentioned physiological signs (i.e., heart activity and blood volume pulse, brain activity, muscle, and neural activity, electrodermal activity, body temperature, and respiratory activity). In this paper, a gamified system in inflammation surveillance and autoimmune disease management is proposed, using the wearable sensor(s) by applying gamification elements in an app to help with the prediction (e.g., information, alerts to health monitoring), management (e.g., progress, points, motivational quotes), and recovery (e.g., rewards, badges, achievements, unlocking resources) in a health crisis.

For individuals to be able to exercise self-care, it is necessary that they have knowledge about the needs of their bodies and know how to act in the face of events that happen for their self-care. Therefore, the proposed app must have a connection with a wearable sensor targeted at people with an autoimmune disease and other users without associated pathologies. Moreover, the app should support (i) the monitoring of physiological symptoms; (ii) the provision of updated information about the topic; (iii) emotional and psychological care; and (iv) suggestions for overcoming the crisis.

To facilitate the development of the concept, a framework becomes necessary, as it is intended to be generic, comprehensive, and flexible enough to serve as a practical guide for the gamification of systems. The 5W2H framework (5 Wh-questions, 2 How) consists of seven ordered steps for the gamification of systems that give rise to its title: ‘Who?’, ‘Why?’, ‘What?’, ‘When?’, ‘How?’, ‘Where?’, and ‘How Much?’ [129], and each of these aims to analyze different aspects to answer each specific question adapted to the context of this paper (Table 3): (i) Who will interact with game elements? (ii) Why does gamification bring benefits to the system? (iii) What will gamification stimulate in its users? (iv) When is the best time to apply each game element? (v) How to apply gamification? (vi) Where to implement game elements? (vii) How much will gamification improve the process of disease management and self-care?

Regarding ‘Who?’, the app targets not only autoimmune patients but also users without diagnosed pathologies, since it is expected to prevent/manage/overcome (any) inflammation crisis and raise awareness. The ‘Why?’ question is easily answered with the motivation and change behaviors factors inherent to gamification. With such elements, the gamified app will tend to be more interesting if used continually and to track changes in behavior. For the ‘What?’ question, the main goal is to stimulate the monitoring symptoms’ frequency, to provide knowledge acquisition about the disease and how to deal with the disease, and ultimately inspire the patients to fight for wellbeing.

Moreover, it is foreseen that the gamification elements will be applied when users are in pre-, during- and post- inflammation phases (‘When?’), specifically by providing information about the disease and notifying the user that health monitoring is needed—e.g., they should take their temperature, see certain inflammatory signs, etc.—by presenting a progress bar with points and motivational quotes to help with the disease’ management and giving badges and skills acquisition for overcoming the crisis by fulfilling achievements and unlocking resources (‘How?’). The gamification system will be implemented in an app for smartphones and smartwatches (‘Where?’), as it is believed that this will improve not only the app, but also the frequency of use, which provides better self-care.

The ideal scenario would be the communication and synchronization of the app with a wearable sensor that provides the biofeedback of ECG and EDA which is already available on the market. It is important that it is able to monitor optical and electrical heart rate, respiratory rate, blood oxygen, blood pressure, restlessness, body temperature, and sleep. The inclusion of brain, muscle, and nerve activity may not be viable, since there is no current affordable offer on the market; however, the app should be capable of providing awareness of those physiological signs through alerts and information. We suggest the use of smartwatches or wristbands for prolonged use and better comfort; thus, the Apple Watch Series 7, Fitbit Sense, Whoop 4, HealBe GoBe2, ZeNeo+, and Voltmi Smart Watch may be suitable for the purpose, since they are able to monitor the intended biofeedback, have different price ranges, and are compatible with Android and/or iOS.

An important aspect to bear in mind when dealing with (confidential) health data, is the need for the implementation of a security layer for data usage control, data administration, service subscriptions, data transfer, data access control, and identity protection. Thus, it is important to ensure [130]: (i) the confidentiality of data that allows the information exchanged to be understood only by the intended entities; (ii) data integrity to ensure that messages exchanged have not been tampered with by third parties; (iii) authentication to ensure the entities involved in any transaction are who they claim to be; (iv) availability so that the service is not interrupted; (v) authorization of entities (i.e., entities now have the necessary control permissions to perform the operation they request); (vi) up-to-date data; (vii) the nonrepudiation that enables that an entity does not evade responsibilities (i.e., cannot deny an action it has taken); and (viii) direct secrecy (i.e., when an entity leaves the network, it will not understand the communications exchanged after it leaves) and reverse secrecy (i.e., any new entity that joins the network will not be able to understand the communications that were exchanged before entering the network).

Additionally, existing application programming interfaces (API) may be used for Bluetooth connectivity that enables (i) the start pairing with a new device, (ii) the control pairing flow via user consent/input and the confirm pairing for inbound and outbound pairing requests, (iii) the establishment of profile connections with a remote Bluetooth device, (iv) the transfer of data to and from other devices, and (v) the communication with wearable sensors that have biofeedback.

Regarding the implementation requirements, it is important to guarantee, in advance, the credibility of the app and its gamified system with connection to the wearable sensor. To this end, it is crucial to involve medical experts and the potential end-users in all the development and testing processes. Moreover, functionality is also a fundamental aspect for the app to not only be informative, but also to provide support for day-to-day self-care. Thus, the concept was developed using existing hardware, software, and technologies, such as the ones mentioned.

### 5.1. Development of the Prototype

**Figure 3:**
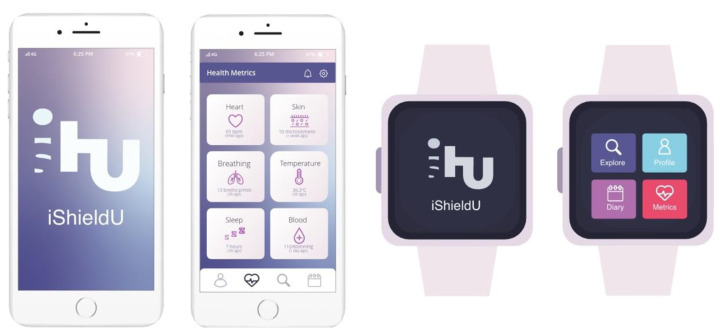
Smartphone app iShU landing and health metrics screen, and smartwatch app iShU landing and dashboard screen (from **left** to **right**).

Based on the literature search, a prototype of a gamified app for inflammation surveillance and autoimmune diseases awareness entitled ‘iShU’ was developed. At the time of writing, the prototyping process was divided into sketching and the first iterative phase for smartphones and smartwatches (Figure 3). It was developed using Adobe Xd and Adobe Illustrator.

The name and logo of the app involved intense brainstorming in order to be short, appealing, and directly linked to the purpose of the app. The authors came down to the brand iShU, which means “I Shield You” and is also the deconstruction of “Iaso Shields You”, since Iaso is a goddess of recuperation of illness and is associated with healing, health, and preventive medicine in Greek mythology.

**Figure 4:**
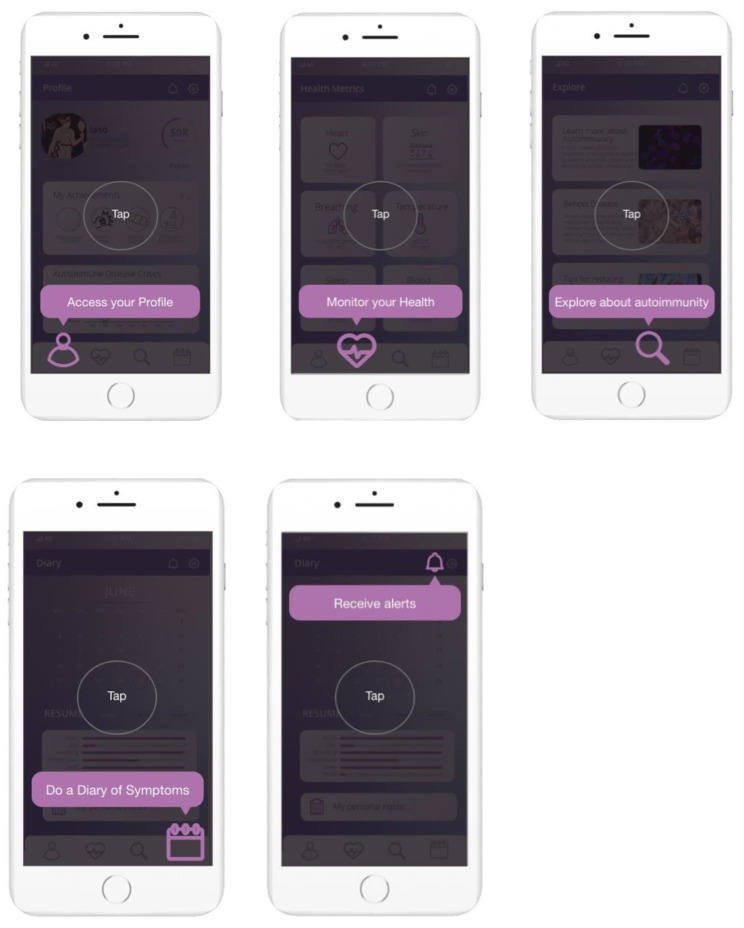
Smartphone app iShU tutorial with main functionalities (from **left** to **right** and **up** to **bottom**).

The main features of this app are (Figure 4): (i) a profile for each user with a name, characterization of identified autoimmune diseases, access to achievements and progress, and a graphic of the overall health in relation to crises and their evolution; (ii) a screen for health metrics with connection to the wearable device, where the user can monitor heart, skin, breathing, temperature, sleep, and blood values; (iii) an explore screen, where the user can find recent and pertinent topics about autoimmunity, a description of each existing autoimmune disease, and several tips to reduce inflammation; (iv) a diary of symptoms with a calendar to add inputs or check previous results and also a notepad to complement the measurements with additional and notes that the user may find relevant; and (v) a notification system with alerts for health measuring, progress feedback, and motivational quotes according to the health status.

The predominant font used in the prototype is Open Sans for its sans serif characteristic and easiness to read. Regarding the color, the use of purple shades and variations of pink and magenta occur because of its psychological meaning—is a rarely occurring color in nature, which combines the calm stability of blue and the fierce energy of red, being often seen as having sacred meaning. Moreover, the purple color is associated with ambition, dignity, independence, and vitality, and pink is linked with relaxation, acceptance, contentment, and a neutralizer of disorder.

### 5.2. Prototype Testing and Evaluation

The authors recruited 15 evaluators, by convenience and snowball sample, who were people with diagnosed autoimmune diseases, cancer patients, health professionals, and information and communication technologies (ICT) experts. The following table (Table 4) presents the sample characterization based on demographics. The recruitment of these participants followed the conditions criteria of being groups of people with a weakened immune system, and/or health professionals, and/or ICT specialists, in order to detect usability problems in the prototype. Furthermore, to respect participants’ privacy, a code was assigned as FIPE (i.e., First Iteration Phase Evaluator) followed by a number.

After recruiting the evaluators, a consent form was provided and a semi-structured interview was conducted as an introduction to the preliminary app testing; then a Adobe Xd link was shared with the participants as well as a script to test the app flow; finally, the participants were asked to answer a survey containing the Nielsen’s 10 heuristics [131] for usability design and a SWOT analysis (i.e., strengths, weaknesses, opportunities, and threats). The semi-structured interview contained demographic questions for sample characterization and a set of questions regarding (i) diagnosed diseases/maladies; (ii) ways of health monitoring and wearable usage; (iii) current gaps in mHealth market; and (iv) desires/expectations for an ideal surveillance app.

At the completion of the characterization of the sample, 20% of the participants (*n* = 3) did not use wearables for health monitoring, measuring their vital signs only when they went to the doctor or pharmacy. Moreover, the remaining participants used smartphones (20%, *n* = 3), smartwatches or wristbands (53%, *n* = 8), chest straps (13%, *n* = 2), GPS sensors (20%, *n* = 3), and glucose monitors (7%, *n* = 1).

With regards to the current gaps in mHealth market, 33% (*n* = 5) stated that most mHealth focuses on basic health metrics instead of other parameters and chronic diseases (other than diabetes and cardiac impairments). Of the participants, 20% also mentioned that these apps are not captivating and do not correspond to the actual users’ context. Moreover, 27% (*n* = 4) complained that they do not have reminders for frequent use and their symptom history is not registered. Additionally, it was also mentioned that there is an interconnect failure between mHealth with healthcare professionals; a shortcoming with urgency mechanisms and customization; a lack of support in post-diagnosis and stress management throughout the course of the disease; missing previous evaluation and validation by experts before its launch on the market; and the tendency to not be accessible for all users due to being expensive and not inclusive.

Participants also shared their desires in a mHealth app, declaring they would like to have the possibility of (i) quickly monitoring all vital signs; (ii) having patient–doctor and patient–patient channels; (iii) having alerts and outreach for app usage, health monitoring, and altered values; (iv) pairing with existing smartwatches; (v) being provided self-care tips and updated information; (vi) having motivators that trigger continued use; (vii) having a repository of symptomatology; and (viii) having an accessible app, both in price and digital inclusion.

After the interview, the iShU app concept was presented, and the participants were asked to test the Adobe Xd link according to the heuristics: (i) H1—visibility of system status; (ii) H2—match between system and the real world; (iii) H3—user control and freedom; (iv) H4—consistency and standards; (v) H5—error prevention; (vi) H6—recognition rather than recall; (vii) H7—flexibility and efficiency of use; (viii) H8—aesthetic and minimalistic design; (ix) H9—help users recognize, diagnose, and recover from errors; and (x) H10—help and documentation. It is important to note that a script was made available to ensure that all evaluators explored the app functionalities in the same way; the heuristics were explained and detailed before the app test and completed according to a five-point Likert scale (1—usability catastrophe; 2—a big issue that has a profound impact on usage and influences the experience; 3—a minor issue that has a small impact on usage and slightly influences the experience; 4—an aesthetic problem that has no impact on use; 5—no problems found). Finally, the participants completed the survey, with regard to the heuristics and the SWOT analysis, to identify the app’s strengths, weaknesses, opportunities, and threats.

The following section presents the evaluation results and their discussion.

## 6. Evaluation and Discussion

This paper has presented the complete process of the literature search, conceptualization, and first iterative testing of a gamified app system with a wearable connection for inflammation detection in autoimmune diseases management.

The practice of self-care helps individuals to monitor their health, especially in the case of chronic diseases, as manifested in autoimmunity. In fact, mHealth technology, combined with gamification, can help the process of behavior change and self-care. Nonetheless, it is important to use wearable sensors as aids in the collection of physiological data since these devices are more suitable for health monitoring than smartphones [77].

Since creating a new wearable device entails unbearable costs and resources, adopting an app’s software to connect to an already commercialized smartwatch becomes more viable. This could increase the quality and validity of a wearable device, as well as allowing a smart integration with gamified mHealth systems. Moreover, the communication between the wearable and the app is a key point of the concept presented in this study. It needs to be functional to ensure the transfer of sensor data to the smartphone via Bluetooth, avoiding data reading and storage errors.

Moreover, heart rate, respiratory rate, blood oxygen, blood pressure, restlessness, body temperature, and sleep are considered, by the authors, to be relevant physiological signs that provide easier inflammatory interpretation. This biofeedback is already monitored by the suggested smartwatches with connections to smartphone apps; thus, it is anticipated that the adaptation and transfer of data to iShU will be feasible.

According to the evaluation of iShU (Table 5), it had an overall median of 4.5 from the 5-point Likert scale. In detail, the recruited evaluators agreed that iShU is an app able to demonstrate its purpose of inflammation surveillance for autoimmune disease management (H1) in a simple and easy way for every user (H2), by providing tutorials for usage learning (H6) and by having a coherent aesthetic design with concise and pertinent information (H8, H10)—where the evaluation was 4.7 and 4.8, respectively. Moreover, H3, H4, H5, and H7 also achieved good scores of 4.3 and 4.4, showing a general agreement on freedom of use, consistency between text, icons and images, error prevention because of a coherent app flow, and accessibility to a broad range of users.

In contrast, the heuristic that generated greater disagreement was “H9—Help and solutions are offered to resolve possible usage errors” (i.e., help users recognize, diagnose, and recover from errors), ranging from 3 to 5. Such disagreement may have happened due to the lack of understanding that arrows to go back and x to eliminate actions were available in several screens with the function of allowing the user to avoid errors. However, thanks to this evaluation, it was possible to perceive that error messages can be introduced at some points of app use.

Furthermore, the participants found the strengths, weaknesses, opportunities, and threats of the app and its inherent concept, which allowed the acknowledgment of the potential of iShU. Regarding the strengths, participants attributed greater relevance to the positive aspects of the app, in which 87% (*n* = 13) stated that the design was appealing, soothing, beautiful, and different from other health apps; 80% (*n* = 12) highlighted the fact that iShU is easy to use because of its simplicity and intuitiveness; 67% (*n* = 10) also appreciated that the app contained a range of symptomatology metrics and information that enables the keeping of a diary as an adjunct to symptoms; moreover, participants pointed out gamification elements (e.g., progress, alerts, motivational quotes), name symbology, innovation and integration for both smartphone and smartwatch, and being dependable as strong features for health monitoring. Some of which are sustained by the following sentences:

*“The application is appealing and very intuitive, promotes healthy habits, allows monitoring of several important indicators and reminds patients about the care they must comply with.”*

—FIPE3

*“[IshU strength is] being an app that covers a wide range of autoimmune diseases, since the existing ones tend to be oriented only to heart, musculoskeletal or diabetes problems.”*

—FIPE6

*“It addresses the priority for patients with systemic/chronic diseases, namely allowing the recording of symptoms in a diary format.”*

—FIPE7

*“Focuses on inflammation key points for a wide range of autoimmune diseases”*

—FIPE8

*“It is a complete app with a value-added purpose, which will bring great expectations to users diagnosed with autoimmune diseases. (…) The existence of the various elements from the concept of diagnosis to the stored reports is very important for an assessment of the general situation of the users, whether carried out by themselves or by healthcare professionals.”*

—FIPE15

Nevertheless, some weaknesses were also identified, such as lack of icons’ identification with text (20%, *n* = 3); that users without some digital skills may find it not so intuitive; usage dependent on a smartwatch; the lack of a social component; the description and evidence of the autoimmune disease; and not monitoring health metrics automatically.

Several opportunities were emphasized: (i) the possibility of exporting all the health data and providing it to a doctor; (ii) the connection between patient and healthcare services; (iii) medication management and reminder; (iv) geolocation to health facilities; (v) being an official app recommended by the health care community; (vi) have a social media/community and forum; (vii) be translated to several languages; and (viii) add features such as the possibility of taking photos of visible symptoms to complete the diagnosis. However, threats of indirect competition with an already strong presence in the mHealth market, as well as ethical issues in data protection and sharing were also pointed out.

Given that the most monitored autoimmune diseases are diabetes and rheumatoid arthritis, there is a gap, as well as an opportunity, in support for other autoimmune diseases, with a special focus on systemic ones that have greater representation of inflammation signs. As such, the authors developed a concept based on the 5W2H framework, since they fit well for the starting point of brainstorming and initial development.

Having considered the overall semi-structured interviews and results from the app testing, it is possible to conclude that iShU have the potential to overcome the current hiatus in the mHealth market and fulfill some of the evaluators’ expectations and desires. Concretely, the app fills some current gaps since it managed to (i) apply a broad range of health metrics to include diverse parameters for inflammation surveillance; (ii) recall frequent use; (iii) record the patients’ history through the symptom diary and summary of inflammatory crises; and (iv) provide support in pre-, during-, and post-inflammatory crises with motivational quotes and calls-to-action. Additionally, iShU was able to reach the following users’ desires: (i) monitor several vital signs with only one app and a wearable sensor; (ii) provide alerts and notifications for continuous app usage, health monitoring, and altered values; (iii) the possibility of pairing with existing smartwatches that are able to include the selected health metrics; (iv) providing self-care tips and updated information about the topic; (v) the use of motivator triggers (e.g., points, badges, motivational quotes) for the continued use of iShU; (vi) a diary that serves not only as a repository of symptomatology, but also a graphic of inflammatory crises’ evolution; and (vii) an accessible app designed to be simples and effective, being digital inclusive.

Following Schmidt-Kraepelin and colleagues’ taxonomical classification [115], this concept and further iterative phases are intended to have: (i) direct concept to user communication via textual and audio outputs; (ii) a static self-selected identity portrayed by a nickname and a picture; (iii) internal rewards; (iv) patients of autoimmune diseases as the target group, but admitting other users without diagnosis; (v) supportive collaboration with linkage to forums and/or social networks; (vi) self and externally set goals; (vii) an expected episodical narrative with different stages associated and a partial users’ progress reset after pre-, during-, and post- inflammation crises; (viii) positive reinforcement with a focus on current and future successes; (ix) behavioral and attitude change as persuasive intent; (x) independent level of integration, as the health monitoring and self-care could be done without the presented proposal; and (xi) progressive user advancement. It is not planned to include the competition gamification element in the app integration.

Furthermore, the study and app development and implementation, should follow and safeguard ethics and deontology in privacy, security, and confidentiality when dealing with health data. All the participants (i.e., experts and potential end-users) signed a written informed consent before participation, stating that they (i) agreed to contribute voluntarily to the research, (ii) were informed of the procedures, (iii) understood the conditions of participation in the research, (iv) authorized the recording and capture of images and audio for research purposes, and (v) permitted the contact via e-mail by the authors for the purposes of the research.

## 7. Final Considerations

In a nutshell, this study enabled further extent current knowledge on the linkage between inflammation and autoimmunity (providing an overview of some autoimmune diseases), the usage of wearable devices in healthcare (detailing the physiological signs detected with different types of sensors), and the relationship established between gamification and mHealth. Furthermore, by crossing these data, the authors presented a concept for gamifying autoimmune disease management.

The immune system is the body’s defense through the production of antibodies and certain types of cells, such as lymphocytes. One of its functions is to protect from invading microorganisms, such as viruses and bacteria, and from diseases in general. Under normal conditions, the immune system does not attack the body’s own cells; however, there are cases in which there is an anomaly, where the immune system mistakes its own cells for invading agents and attacks them instead of protecting them. From these situations, autoimmune diseases may arise that can affect an individual in a localized (i.e., organ-specific) or generalized (i.e., systemic) way. Symptoms of autoimmune diseases can vary greatly in type and degree, with some people experiencing milder manifestations of the disease, while others may have severe complaints. For example, a common sign of the autoimmune disease is inflammation, which manifests as redness, localized heat, pain, and swelling.

It is worth noting that health care is a recurring topic in society, every day there are several advances, new techniques, and medicines, so it is necessary to create means to encourage the citizen to use them. Advances in communication technologies and information systems allow the development of systems capable of sending data, e.g., of temperature, heart rate, and blood pressure. Wireless health applications, using a network of wireless sensors can help many users by allowing constant and noninvasive monitoring without regular medical follow-ups.

Gamification can be applied in the health sector through the adoption of policies and strategies that can influence people’s lifestyles effectively and establish some level of responsibility for the management of personal health. Through this technique, a low-cost relationship can be established, acting in a preventive way based on individual information to improve health status instead of investing only in the treatment of symptoms. It also presents a favorable approach to overcome loss of interest, to engage users, to increase the quality of healthy behaviors, and to motivate users to utilize mHealth apps for a continued period.

The developed app iShU integrates the connection between smartphone and smartwatch and the requirement of (i) monitoring physiological symptoms—such as heart, skin, breathing, temperature, sleep, and blood—that may identify inflammatory crises (available on the health metrics and user profile); (ii) providing updated and pertinent information about inflammation and autoimmune diseases (available on the explore screen); (iii) providing emotional and psychological care in the feedback of actions and notifications; and (iv) providing suggestions for overcoming the crisis (e.g., “Your values are a little bit off today ☹ Make sure your drink a chamomile tea and get some rest. You will feel better!”). Thus, such requirements are able answer the research question, “What are the design requirements for an application to foster Inflammation Surveillance and Autoimmune Disease Management?”.

This paper presents a few limitations, as the study of autoimmune diseases (especially the systemic ones) is under constant evolution and discovery, and it is difficult to represent all patients with the mentioned and nonmentioned diseases. Moreover, as this study relies on a literature search of topics that were not found in the literature, a range of relevant papers may have not be considered for this study. Furthermore, the developed app only had an initial evaluation with 15 participants; therefore, further evaluations in iterative phases with a bigger sample for more cohesive results is necessary.

Regarding future work, the inclusion of scheduling a doctor’s appointment and medication; the social component to foster a self- and community-care experience; and the integration of photos of the symptoms described may be a positive addition, as they were desired by the participants. Moreover, an iterative participatory design approach, with a bigger sample of experts (e.g., medicine and gamification researchers and doctors), is necessary to move from the concept to the implementation of the digital entity in order to have a user-centered product that can fulfill its purpose. Furthermore, the recurrent evaluation process and analysis of other potential frameworks and the inclusion of extra biofeedback may be needed to guarantee suitability and effectiveness.

Nevertheless, the authors believe that the study reported in this paper may provide insightful knowledge and contribution to the field of medicine (internal, general, family medicine, and rheumatologists), human–computer interaction, psychology, and gamification, as this is a subject with an associated innovative factor. As mentioned before, there is a lack of research and applications within this specific topic of autoimmune diseases; thus, iShU may be a pioneer in such matters. Furthermore, the prototype has been validated in the first iterative test, which guarantees that the concept is relevant and needed, sustaining its further development, implementation, and launch.

## Figures and Tables

**Table 2:** Commercialized wearable sensors to detect physiological signs.

Physiological Sign	Wearable Sensor
ECG and BVP	Empatica E4 wrist band, AutoSense, Cardiosport TP3, BioHarness 3, V800 Polar^®^, BN-PPGED
EEG	Actiwatch 2, MindWave mobile EEG headset, WatchPAT, Emotiv EPOC
EMG	DataLOG
EDA	Empatica E4 wrist band, BN-PPGED, Q-sensor, Shimmer sensor, Moodmetric EDA Ring
Body Temperature	Empatica E4 wrist band, AutoSense
Respiratory activity	AutoSense, SleepSense

**Table 3:** Concept answers based on the 5W2H framework.

Question	Answer
Who?	The app is aimed not only at autoimmune patients but also at users without diagnosed pathologies.
Why?	Motivation and behavior changes.
What?	Frequency in monitoring symptoms; Inspiration to continue to fight and manage the disease; Knowledge acquisition.
When?	Pre-, During-, Post-Inflammation crises.
How?	Pre—information, alerts/notifications, check-in; During—progress, points, motivational quotes; Post—skills, badges, achievements, unlocking resources.
Where?	App for Smartphone and Smartwatch
How Much?	It is expected that this will be the key to help maintain the frequency of health monitoring and reporting, and the motivation to fight the disease, thus improving self-care.

**Table 4:** Sample characterization.

Code	Age	Gender	Occupation	Diagnosed Disease
FIPE1	41	Female	Healthcare professional (nurse)	Hypothyroidism
FIPE2	48	Male	Healthcare professional (nurse)	N/A
FIPE3	47	Female	Researcher in Human–Computer Interaction with expertise in eHealth and digital inclusion	N/A
FIPE4	36	Female	Scholar with expertise in ICT	N/A
FIPE5	26	Female	Freelancer and ICT expert	Hashimoto’s Thyroiditis; Rhinitis, Sinusitis, Asthma, Urticaria, Eczema
FIPE6	47	Male	Healthcare Professional (clinical physiologist)	N/A
FIPE7	23	Female	Scholar with expertise in ICT/UX	Behçet; Allergic Rhinitis
FIPE8	59	Male	Bank Employee	Stomach Cancer
FIPE9	58	Female	Retired	Breast Cancer
FIPE10	27	Female	Scholar with expertise in ICT/UX	Behçet; Lupus Systemic Erythematosus
FIPE11	27	Male	Data analyst	Crohn’s disease
FIPE12	43	Male	Real estate agent	Diabetes Mellitus Type 1
FIPE13	40	Female	Scholar with expertise in eHealth technologies	N/A
FIPE14	48	Male	Healthcare professional (pharmaceutical)	N/A
FIPE15	41	Female	Healthcare professional (nurse)	N/A

**Table 5:** Median of each heuristic evaluation.

Heuristic Detailed Description	Evaluation
H1—Keeps the user up to date on the issue, use and purpose of the iShU app.	4.7
H2—The language used in iShU is understandable even for those who are not knowledgeable about the topic.	4.7
H3—iShU guides the users, but also allows them to use it freely.	4.3
H4—Users do not need to worry if different words/terms/icons/images have the same meaning.	4.3
H5—iShU’s design carefully avoids possible errors or usage problems.	4.4
H6—There is a minimization of the use of users’ memory by the provision of instructions/tutorials.	4.7
H7—iShU can be used by experienced or inexperienced users.	4.3
H8—The design and aesthetic information available on iShU do not interfere with the information of the available content.	4.7
H9—Help and solutions are offered to resolve possible usage errors.	3.9
H10—iShU provides information in a concise way, without getting in the way of the task.	4.8

## Data Availability

Not applicable.
